# Use of Non-pharmacological Therapies in Individuals With Migraine Eligible for Treatment With Monoclonal Antibodies Targeting Calcitonin Gene-Related Peptide (CGRP)-Signaling: A Single-Center Cross-Sectional Observational Study

**DOI:** 10.3389/fpain.2022.935183

**Published:** 2022-07-13

**Authors:** Lucas Rundblad, Christopher Kjaer Cullum, Simona Sacco, Raquel Gil-Gouveia, Derya Uludüz, Thien Phu Do, Faisal Mohammad Amin

**Affiliations:** ^1^Danish Headache Center, Department of Neurology, Rigshospitalet Glostrup, Faculty of Health and Medical Sciences, University of Copenhagen, Copenhagen, Denmark; ^2^Department of Biotechnological and Applied Clinical Sciences, Neurological Institute, University of L'Aquila, L'Aquila, Italy; ^3^Hospital da Luz Headache Center, Neurology Department, Hospital da Luz, Lisboa, Portugal; ^4^Universidade Católica Portuguesa, Institute of Health Sciences, Center for Interdisciplinary Research in Health, Lisboa, Portugal; ^5^Neurology Department, Istanbul University Cerrahpaşa School of Medicine, Istanbul, Turkey; ^6^Danish Knowledge Center on Headache Disorders, Rigshospitalet Glostrup, Glostrup, Denmark; ^7^Department of Neurorehabilitation/Traumatic Brain Injury, Rigshospitalet, University of Copenhagen, Copenhagen, Denmark

**Keywords:** acupuncture, chiropractic, complementary and alternative medicine, headache, migraine, osteopathy, physical therapy, reflexology

## Abstract

**Introduction:**

Accessibility of treatment with monoclonal antibodies targeting the calcitonin gene-related peptide (CGRP) signaling pathway is impeded by regulatory restrictions. Affected individuals may seek out other services including non-pharmacological therapies. Thus, we found it timely to ascertain the use of non-pharmacological therapies in individuals with treatment-resistant migraine eligible for and naïve to treatment with CGRP-signaling targeting monoclonal antibodies.

**Methods:**

We conducted a single-center cross-sectional observational study of patients eligible for and naïve to treatment with monoclonal antibodies targeting CGRP or its receptor. We recorded demographical information (gender, age, educational level, employment status, and income), disease burden (frequency of headache days and migraine days), previous use of preventive pharmacological medications for migraine, and use of non-pharmacological therapies over the past 3 months including frequency of interventions, costs, and patient-reported assessment of efficacy on a 6-point scale (0: no efficacy, 5: best possible efficacy).

**Results:**

We included 122 patients between 17 June 2019 and 6 January 2020; 101 (83%) were women and the mean age was 45.2 ± 13.3 years. One-third (*n* = 41 [34%]) had used non-pharmacological therapy within the past 3 months. Among these participants, the median frequency of different interventions was 1 (IQR: 1–2), the median number of monthly visits was 2.3 (IQR: 1.3–4), mean and median monthly costs were 1,086 ± 1471, and 600 (IQR: 0–1200) DKK (1 EUR = ~7.5 DKK), respectively, and median patient-reported assessment of the efficacy of interventions was 2 (IQR: 0–3).

**Conclusion:**

Even in a high-income country with freely accessible headache services and universal healthcare coverage, there was a non-negligible direct cost in parallel with low satisfaction for non-pharmacological therapies among patients at a tertiary headache center.

## Introduction

Headache disorders constitute a major public health problem as they lead to significant disability worldwide, impair quality of life, reduce productivity, and incur a substantial financial burden on both individuals and economies (1–3). Migraine, specifically, directly affects more than 1 billion people across the world and constitutes a leading cause of disability (1, 2). In Europe, the financial burden of migraine has been estimated at €50 to €111 billion in 2011 (3). Assumingly, these costs are the highest for those with the highest disease burden, i.e., individuals with chronic migraine, and evidence suggests that these individuals often require referral to specialist care (4). These services include treatment with migraine-specific medications that target the calcitonin gene-related peptide (CGRP) signaling pathway and are efficacious and tolerable in patients with treatment-resistant migraine (4, 5). In Europe, monoclonal antibodies (mAbs) against the CGRP ligand or its receptor are the drugs available in this class, but their accessibility is low as regulatory restrictions often limit their use due to high costs (4). Even though some countries provide free access to these therapies if pre-specified eligibility criteria are fulfilled, accessibility is often impeded as these medications are available at a limited number of specialist services. In clinical practice, severely affected individuals often seek out other services including non-pharmacological therapies (5), but data on this perspective is sparse. Thus, we found it timely to ascertain the use of non-pharmacological therapies in individuals with treatment-resistant migraine eligible for and naïve to treatment with CGRP-signaling targeting mAbs in a single-center cross-sectional observational study. In particular, we investigated the associated direct costs and patient satisfaction of non-pharmacological treatments.

## Methods

### Study Overview

We conducted a single-center, cross-sectional observational study. The present study was approved by the regional ethics committee and the Danish Data Protection Agency. All participants provided written informed consent before any assessments. We conducted the study in accordance with the Declaration of Helsinki (6).

### Study Population

Participants eligible for and naïve to treatment with CGRP-mAbs followed at a tertiary headache center (the Danish Headache Center). According to national practice guidelines, participants eligible for treatment with and reimbursement for CGRP-targeting mAbs had a diagnosis of chronic migraine in accordance with the International Classification of Headache Disorders, 3rd edition (ICHD-3) (7) and documented failure based on lack of efficacy or tolerability of at least one antihypertensive and one anticonvulsant that is used for migraine prevention. Exclusion criteria were medication-overuse headache (MOH), as defined in ICHD-3, as this is a criterion for reimbursement for treatment with CGRP-targeting mAbs in Denmark (7). Participants underwent a semi-structured interview on demographical information (gender, age, educational level, employment status, and income), disease burden (frequency of headache days and migraine days), previous use of preventive pharmacological medications for migraine (with no time restrictions), and use of non-pharmacological therapies the past 3 months (both self-referral and by prescription), including frequency of interventions, costs, and patient-reported assessment of efficacy on a 6-point scale (0: no efficacy, 5: best possible efficacy). Non-pharmacological therapies were assessed for the past 3 months to determine current active use.

### Statistical Methods

Continuous and count outcomes are presented using means with SDs. Binary and multinomial outcomes are presented with absolute numbers and percentages. All other data are presented as reported.

## Results

### Sociodemographic and Migraine-Related Characteristics

A total of 122 patients were included in the study between 17 June 2019 and 6 January 2020. As shown in Table 1, 101 (83%) were women, 21 (17%) were men, the mean age was 45.2 ± 13.3 years, 34 (28%) had a university degree, 75 (61%) were employed full time, part-time, manager or self-employed, and 25 (20%) had an annual income >400,000 DKK (~53,000 EUR). The baseline means monthly headache days was 22.3 ± 5 days, and the baseline mean monthly migraine days was 17.2 ± 6.3 days. One-fourth (31 [25%]) of the population was using any pharmacological preventive medication at the time. All patients had discontinued the use of at least two preventive medications for migraine due to lack of efficacy or tolerability; the median number of previous preventive medications was 7 (IQR: 5–8).

**Table 1:** Sociodemographic and migraine-related characteristics of participants using and not using non-pharmacological therapies.

	**Total population** ***n* = 122 (100%)**	**Use of non-pharmacological therapy in the past 3 months** ***n* = 41 (34%)**	**No use of pharmacological therapy in the past 3 months** ***n* = 81 (66%)**
**Women**, *n* (%)	101 (83%)	37 (90%)	64 (79%)
**Age (years)**, mean (SD)	45.2 (±13.3)	43.4 (±13.1)	46.1 (±13.3)
**No. of monthly headache days**, mean (SD)	22.3 (±5.0)	22.6 (±4.9)	22.2 (±5.1)
**No. of monthly migraine days, mean (SD)**	17.2 (±6.3)	16.8 (±6.2)	17.4 (±6.3)
**Current use of preventive medications**, *n* (%)	31 (25%)	15 (37%)	16 (20%)
**Previous preventive medications**, median (IQR)	7 (5–8)	6 (5–8)	7 (5–8)
**Education**, *n* (%)			
Less than high school	5 (4%)	2 (5%)	3 (4%)
Short–cycle higher education	11 (9%)	4 (10%)	7 (9%)
Medium-cycle higher education (programme/qualification)	67 (55%)	16 (39%)	51 (63%)
University degree	34 (28%)	17 (41%)	17 (21%)
Other	4 (3%)	2 (5%)	2 (2%)
**Employment status**, *n* (%)			
Employed full time	42 (34%)	10 (24%)	32 (40%)
Employed part time	15 (12%)	6 (15%)	9 (11%)
Manager	11 (9%)	4 (10%)	7 (9%)
Self-employed	7 (6%)	3 (7%)	4 (5%)
Retired	14 (11%)	3 (7%)	11 (14%)
Long-term disability or sick leave	7 (6%)	2 (5%)	5 (6%)
Student	11 (9%)	4 (10%)	7 (9%)
Not employed	12 (10%)	7 (17%)	5 (6%)
**Annual personal income (DKK)**^**1**^, *n* (%)			
<100.000	15 (12%)	8 (20%)	7 (9%)
100.000–200.000	24 (20%)	4 (10%)	20 (25%)
200.001–300.000	28 (23%)	13 (32%)	15 (19%)
300.001–400.000	29 (24%)	7 (17%)	22 (27%)
>400.000	25 (20%)	9 (22%)	16 (20%)

*^1^One EUR is ~7.5 DKK. DKK, Danish krone; SD, standard deviation*.

### Use of Non-pharmacological Therapies

The proportion of patients who had at least one non-pharmacological treatment for migraine in the previous 3 months was one-third (*n* = 41 [34%]) (Table 1). Among this one-third who had used a non-pharmacological treatment, an intervention with physical therapy (*n* = 17 [41%]) or massage (*n* = 17 [41%]) was most common. Other interventions included reflexology, (*n* = 7 [17%]), acupuncture (*n* = 6 [15%]), chiropractic (*n* = 4 [10%]), craniosacral therapy (*n* = 3), osteopathy (*n* = 3 [12%]), and others (*n* = 4 [12%]). Others included Body Self Development (Body SDS, *n* = 1), freeze gel (*n* = 1), mindfulness (*n* = 1), and neuromodulation device (*n* = 1). The median frequency of different non-pharmacological interventions was 1 (IQR: 1–2), the median number of monthly visits was 2 (IQR: 1–4) visits, and the mean and median monthly costs were 1,086 ± 1,471 and 600 (IQR: 0–1200) DKK (1 EUR = ~7.5 DKK), respectively, and the median patient-reported assessment of the efficacy of interventions was 2 (IQR: 0–3). Twelve patients (29%) out of 41 patients who used non-pharmacological therapies did not pay for their inventions.

## Discussion

In a population of individuals with chronic migraine eligible for and naïve to treatment with mAbs targeting CGRP or its receptor at a tertiary headache center, one-third had used non-pharmacological therapies within the past 3 months.

Several non-pharmacological therapies are used in clinical practice for migraine, but there is limited evidence of the clinical benefits of these interventions for chronic migraine (5). While there is some evidence for neuromodulation and biobehavioral therapies, e.g., cognitive behavioral therapy, there is less evidence for the use of physical therapy for chronic migraine (5). Yet, physical therapy (alongside massage) was the most common intervention among users of non-pharmacological therapies. Musculoskeletal symptoms are common both outside and during migraine attacks in patients (8–11). Consequently, it has been suggested that interventions targeting these factors may provide clinical benefits, which provide a possible explanation for its popularity in this cohort. Another possible explanation is that physical therapy is often suggested as an adjunct therapy (5). However, a randomized clinical trial did not report any further gains from physical therapy as an adjunct to standard care (12), and a meta-analysis of controlled trials found that these interventions did not affect the frequency and intensity of attacks, albeit there was possibly a reduced duration of migraine attacks (13). Similarly, there is a low to very low level of evidence for other popular interventions in this cohort (5, 14).

There was a non-negligible direct cost of ~1,000 DKK (~133 EUR) per month in patients who had used non-pharmacological treatments within the past 3 months. With a median hourly wage of 218 DDK (~29 EUR) in 2012, this represents a net cost of ~5 h or 14% of a standardized Danish 37-h workweek (15). Of note, the majority of the cohort had an annual income lower than average (<400 DKK; ~53,000 EUR) (Table 1). (15) and the relative cost is, therefore, higher for this population. This is despite the welfare system of Denmark providing free access to healthcare providers, subsidization of patient fees for both pharmacological and non-pharmacological therapies, and, potentially, universal coverage for all its residents. Consequently, the true direct (and indirect) costs are almost certain to be higher – and perhaps much higher – than in our sample (many patients did not pay for their non-pharmacological treatments), and direct costs have been estimated to constitute 7% of the overall financial burden of migraine in Europe (3). When inquired to rate the efficacy of their non-pharmacological treatments, patients in this cohort ranked the efficacy of interventions in the lower end of the scale, and these findings may reflect that patients refractory to treatment with standard care are more likely to seek out and pay for other services despite the marginal benefits. However, the number of previous preventive medications between users and non-users of these services appear comparable. In addition, there does not appear to be a clear pattern related to the reported sociodemographic factors, but further data are needed to clarify this aspect.

The present study has some limitations. First, we did not inquire about the lifetime use of non-pharmacological interventions, which could have provided further insight into the non-users in our cohort, e.g., this sub-population may not have had any active use of other popular non-pharmacological treatments, e.g., dietary interventions, due to previous failed attempts in the past. Second, the design of the study was a priori descriptive with a relatively modest sample size in combination with averaging patient-reported outcomes and costs of different interventions, which limits regression modeling; therefore, more detailed investigations are merited to determine potential influencing factors.

## Conclusion

Even in a high-income country with freely accessible headache services and universal healthcare coverage, there is a non-negligible direct cost for non-pharmacological therapies combined with overall low satisfaction with these therapies amongst patients treated at a tertiary headache center. These findings can be used to support policy-making decisions and incentivize stakeholders to make sensible healthcare policies to improve headache services. It is imperative that we further assess and address factors that influence treatment patterns.

## Data Availability Statement

Data supporting the conclusions of this article is available upon reasonable request and acquisition of necessary permissions.

## Ethics Statement

The studies involving human participants were reviewed and approved by Regional Committee on Health Research Ethics (RegionH). The patients/participants provided their written informed consent to participate in this study.

## Author Contributions

FA and TD contributed to conception and design of the work. LR and CC contributed to acquisition of data for the work. LR, CC, TD, and FA contributed to analysis of data for the work and wrote the first draft of the manuscript. All authors contributed to interpretation of data for the work, contributed to critical revision of the work for important intellectual content, and read and approved the final manuscript.

## Conflict of Interest

SS reports grants, personal fees and/or non-financial support from Allergan, Novartis, Teva, Eli Lilly, AstraZeneca, Abbott, Medscape, Pfizer, Bayer, Medtronic, Starmed, Bristol-Meyer-Squibb, Daiichi-Sankyo, Lundbeck, Uriach and Neurodiem Ology Medical Education. RG-G reports Honoraria for conferences, consulting or educational activities: Novartis, Allergan/ Abbvie, Teva, Lilly, Lundbeck, Tecnifar, Pfizer, FLOAT, CMBE. Research grants: Fundação para a Ciência e Tecnologia (project 29675, MigN2Treat, 02/SAICT/2017), Novartis-Sociedade Portuguesa de Cefaleias and Learning-Health, Luz Saúde (Research group LiON, Luz Innovation on Neurosciences). DU reports grants, personal fees and/or non-financial support from Allergan, Novartis, Teva, Eli Lilly. FA has received honoraria and personal fees from Teva, Lundbeck, Novartis, Eli Lilly for lecturing or participating in advisory boards. The remaining authors declare that the research was conducted in the absence of any commercial or financial relationships that could be construed as a potential conflict of interest.

## Publisher's Note

All claims expressed in this article are solely those of the authors and do not necessarily represent those of their affiliated organizations, or those of the publisher, the editors and the reviewers. Any product that may be evaluated in this article, or claim that may be made by its manufacturer, is not guaranteed or endorsed by the publisher.

## Abbreviations

CGRP: calcitonin gene-related peptide
DKK: Danish krone
ICHD-3, International Classification of Headache Disorders: third edition
IQR: interquartile range
mAb: monoclonal antibody
SD: standard deviation.
